# Trastuzumab Deruxtecan‐Induced Myotoxicity: First Case Report About Myopathy in Patient With Normal Body Composition and Clinical Insights

**DOI:** 10.1111/1759-7714.70168

**Published:** 2025-09-26

**Authors:** Zhen Qiao, LiuDan Li, Hong Wang, ShuHui Dai, LiTong Ye

**Affiliations:** ^1^ Department of Breast Surgery Zhuhai Center For Maternal and Child Health Care (Zhuhai Women and Children's Hospital) Zhuhai China; ^2^ Department of Nursing Zhuhai Center For Maternal and Child Health Care (Zhuhai Women and Children's Hospital) Zhuhai China; ^3^ Hospital Infection Control Department Zhuhai Center For Maternal and Child Health Care (Zhuhai Women and Children's Hospital) Zhuhai China

**Keywords:** creatine kinase, glutathione, magnesium isoglycyrrhizinate, myotoxicity, trastuzumab deruxtecan

## Abstract

Trastuzumab deruxtecan (T‐DXd) significantly improves outcomes in HER2‐positive or low metastatic breast cancer (MBC), but systematic documentation of its myotoxicity is lacking. A 45‐year‐old woman with HER2‐low MBC and normal body composition (BMI 22.9 kg/m^2^, visceral adipose area [VAT] 69.1 cm^2^) developed T‐DXd myopathy. She experienced dysphagia, Grade III myalgia, and creatine kinase (CK) peak of 1755 U/L, with MRI confirming lumbar subcutaneous edema and paraspinal muscle swelling. T‐DXd was discontinued. Supportive therapy included hydration, urine alkalization by sodium bicarbonate, glutathione, and magnesium isoglycyrrhizinate. By Day 8, CK decreased to 539 U/L with myalgia improvement. After 13 days off therapy, CK rebounded to 1735 U/L with Grade III myalgia, which resolved upon reinitiating support. This case report presents the first documented instance of severe T‐DXd‐related myopathy in a patient with normal body composition. The observed case outcomes suggest that the combination of glutathione and magnesium isoglycyrrhizinate could potentially reduce CK levels and alleviate T‐DXd‐associated muscle pain. However, the observed clinical efficacy is based on an individual case. Extrapolation of these clinical outcomes requires large‐scale randomized controlled trials with rigorous covariate adjustment.

## Graphical abstract

A 45‐year‐old woman with HER2‐low MBC and normal body composition (BMI 22.9 kg/m^2^, visceral adipose area [VAT] 69.1 cm^2^) developed Trastuzumab deruxtecan (T‐DXd) myopathy. She experienced dysphagia, Grade III myalgia, and creatine kinase (CK) peak of 1755 U/L, with MRI confirming lumbar subcutaneous edema and paraspinal muscle swelling. T‐DXd was discontinued. Supportive therapy included hydration, urine alkalization by sodium bicarbonate, glutathione, and magnesium isoglycyrrhizinate. By Day 8, CK decreased to 539 U/L with myalgia improvement. After 13 days off therapy, CK rebounded to 1735 U/L with Grade III myalgia, which resolved upon reinitiating support.

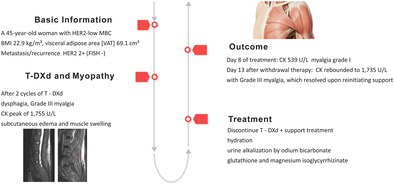

## 1 Introduction

Trastuzumab deruxtecan (T‐DXd) is a novel antibody‐drug conjugate (ADC) targeting human epidermal growth factor receptor 2 (HER2). Results from studies such as DESTINY‐Breast03 [1] and DESTINY‐Breast04 [2] demonstrate that T‐DXd not only significantly improves survival in patients with HER2‐positive advanced breast cancer but also provides clinical benefit to patients with HER2‐low advanced breast cancer from HER2‐targeted therapy [3, 4, 5]. As widespread adoption of T‐DXd occurs, greater emphasis is being placed on its safety profile and the management of associated adverse reactions. Common adverse events include infusion‐related, gastrointestinal, hematological, respiratory, cardiovascular, and hepatic adverse events [6], however, musculoskeletal toxicity has not been systematically reported in the literature. Drug‐induced myopathy refers to muscle tissue damage caused by medications, which can lead to rhabdomyolysis and even carry the risk of acute renal failure [7, 8]. Currently, the established treatment strategies for drug‐induced myopathy are immunosuppressive therapy, such as glucocorticoids, which represents the primary therapeutic approach [9]. As limited efficacy in non‐immune‐mediated myopathy and high‐dose corticosteroids may interfere with antitumor efficacy [10], there is an urgent need for non‐immunosuppressive therapeutic options for drug‐induced myopathy. In this article, we systematically describe a patient with HER2‐low recurrent metastatic breast cancer who developed severe T‐DXd myopathy for the first time. We found that glutathione combined with magnesium isoglycyrrhizinate effectively reduced creatine kinase (CK) levels, proposing a novel therapeutic strategy for drug‐induced myopathy.

## 2 Case Report

A 45‐year‐old patient with metastatic breast cancer, without prior history of myopathy, underwent modified radical mastectomy for left breast cancer in November 2017 (pT2N1M0, stage IIB). Immunohistochemistry demonstrated estrogen receptor (ER)‐positive, progesterone receptor (PR)‐positive, and HER2‐positive (IHC 3+) status. Postoperatively, she received 4 cycles of epirubicin plus cyclophosphamide, followed by 4 cycles of paclitaxel with trastuzumab as adjuvant therapy. After completing radiotherapy, she continued 1‐year maintenance therapy with trastuzumab plus toremifene, subsequently maintaining toremifene monotherapy for 2 years.

From December 2021 to April 2024, she developed metastatic disease in the left supraclavicular lymph nodes followed by left chest wall recurrence. Immunohistochemistry revealed: ER (40%, positive), PR (10%, positive), HER2 (IHC 2+ with negative FISH). Subsequent treatments included: 6 cycles of chemotherapy with carboplatin plus albumin‐bound paclitaxel, endocrine therapy with fulvestrant plus exemestane and goserelin, 8 cycles of chemotherapy with liposomal paclitaxel, maintenance therapy with capecitabine. Drug‐related adverse reactions primarily presented as myelosuppression, which reached grade IV severity at its worst. Creatine kinase (CK) levels remained within normal limits, and no manifestations of myalgia were observed.

In May 2024, she developed disease progression with metastasis to the right supraclavicular lymph node and cervical lymph nodes. Immunohistochemistry revealed ER (1%+), PR negative, HER2 (IHC 2+ with negative FISH). The physician initiated chemotherapy with eribulin. However, CA 15‐3 levels continued to rise, and the lymph nodes increased in size compared to previous scans. Consequently, the treatment regimen was changed to trastuzumab deruxtecan (T‐DXd).

**FIGURE 1:**
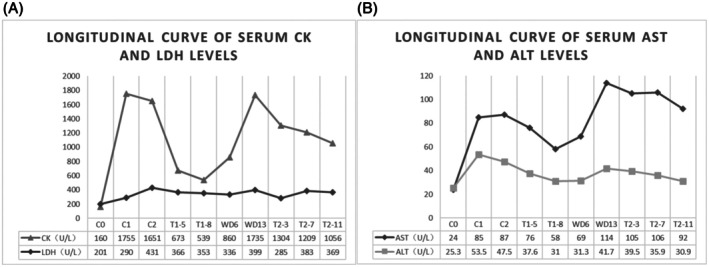
(A) Longitudinal monitoring of serum CK and LDH levels. (B) Longitudinal monitoring of serum AST and ALT levels. Abbreviations: C0: pre‐dose baseline of T‐DXd; C1: post‐cycle 1 administration of T‐DXd; C2: post‐cycle 2 administration of T‐DXd; T1‐5: Day 5 of initial supportive therapy; T1‐8: Day 8 of initial supportive therapy; WD6: Day 6 after withdrawal of supportive therapy; WD13: Day 13 after withdrawal of supportive therapy; T2‐3: Day 3 of resumed supportive therapy; T2‐7: Day 7 of resumed supportive therapy; T2‐11: Day 11 of resumed supportive therapy.

Prior to T‐DXd initiation, the patient's baseline CK level was 160 U/L. Following the first cycle of T‐DXd (5.4 mg/kg), CK rose significantly to 1755 U/L (Figure 1A), LDH increased to 290 U/L (Figure 1A), and aspartate aminotransferase (AST) was elevated to 85 U/L (Figure 1B). N‐terminal pro‐B‐type natriuretic peptide (NT‐proBNP) and high‐sensitivity troponin T (hsTnT) levels remained within normal limits (Table 1). The patient experienced bilateral lower limb myalgia and exhibited muscle strength of grade II (MRC scale) in both upper and lower limbs.

**TABLE 1:** BNP and hsTnT ng/L during therapy.

Parameter	Baseline	C1	C2	T1‐8	WD13
NT‐proBNP (pg/mL)	—	< 10.00	186.6↑	257.2↑	27.58
hsTnT (ng/L)	—	5.13	—	—	—

Abbreviations: C1: post‐cycle 1 administration of T‐DXd; C2: post‐cycle 2 administration of T‐DXd; T1‐8: Day 8 of initial supportive therapy; WD13: Day 13 after withdrawal of supportive therapy.

**FIGURE 2:**
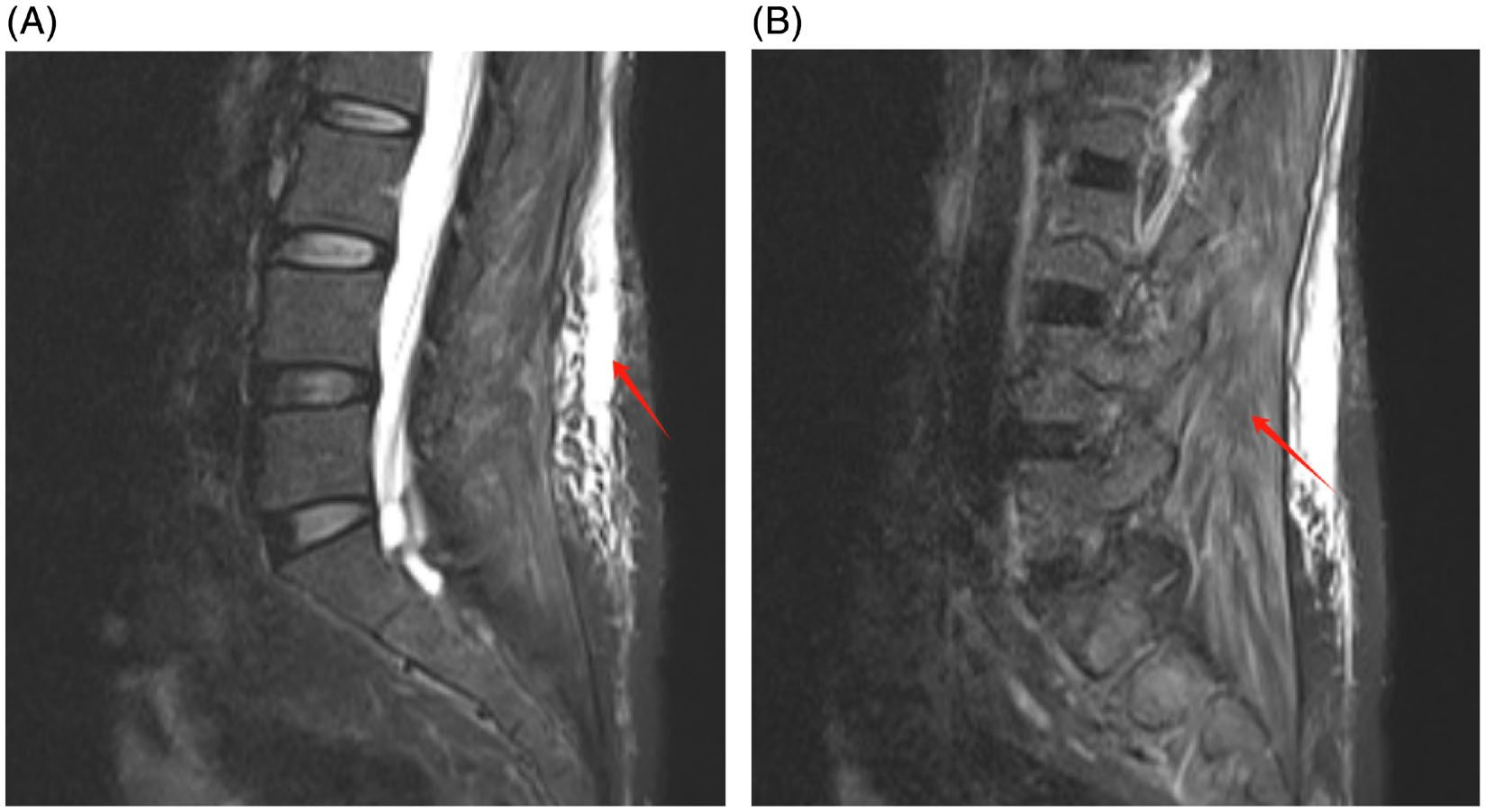
Axial T2‐weighted MRI of the lumbosacral region. (A) High‐signal edema in subcutaneous soft tissues (arrow). (B) Swelling of paraspinal muscles (arrow).

For the second cycle, the T‐DXd dose was reduced to 4.4 mg/kg. One week after administration, the patient developed dysphagia and generalized myalgia, predominantly in the lower limbs (Grade 3 myalgia). CK peaked at 1651 U/L (Figure 1A), LDH increased to 431 U/L (Figure 1A), and AST rose to 87 U/L (Figure 1B). Magnetic resonance imaging (MRI) revealed diffuse subcutaneous edema (Figure 2A) and muscle swelling in the lumbar and dorsal regions (Figure 2B).

**FIGURE 3:**
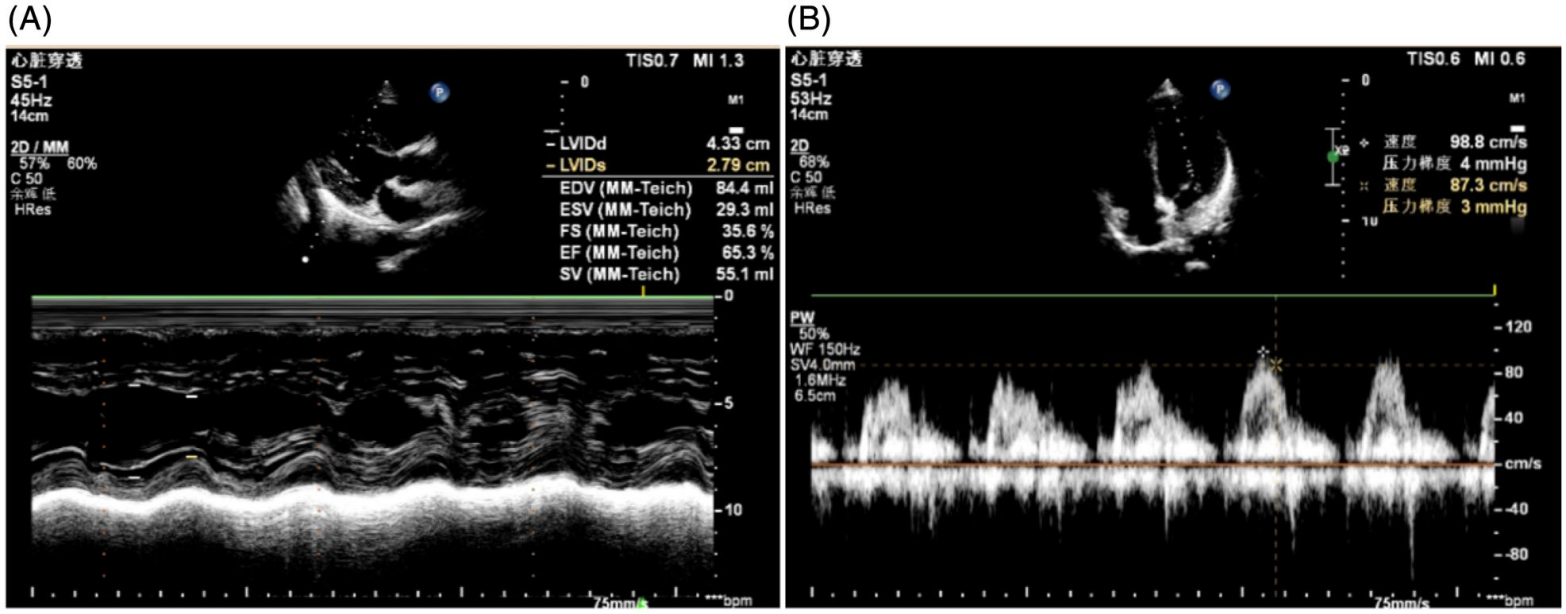
Transthoracic echocardiography with Doppler. (A) Demonstrates left ventricular ejection fraction (LVEF) of 65.3%. (B) Shows mitral inflow velocities—E‐wave 98.8 cm/s, A‐wave 87.3 cm/s, E/A ratio = 1.13.

Body composition analysis showed normal findings: Body Mass Index (BMI) 22.9 kg/m^2^, skeletal muscle mass 23.9 kg, and visceral fat area 69.1 cm^2^. Transthoracic echocardiography demonstrated normal left ventricular ejection fraction (LVEF 65.3%) (Figure 3A) and normal diastolic function (E/A ratio = 1.13) (Figure 3B). Investigations including antinuclear antibody (ANA) profile (Table 2), rheumatological markers, and inflammatory markers were all unremarkable/within normal limits.

**TABLE 2:** Antinuclear antibody (ANA) profile.

Antibody	Result	Reference
Anti‐nucleosome	Negative	Negative
Anti‐dsDNA	Negative	Negative
Anti‐histone	Negative	Negative
Anti‐Smith (Anti‐Sm)	Negative	Negative
Anti‐snRNP	Negative	Negative
Anti‐Ro60	Negative	Negative
Anti‐Ro52	Negative	Negative
Anti‐La	Negative	Negative
Anti‐topoisomerase I (Scl‐70)	Negative	Negative
Anti‐centromere (ACA)	Negative	Negative
Anti‐Jo‐1	Negative	Negative
Anti‐ribosomal P	Negative	Negative

T‐DXd was discontinued, and supportive care was initiated: Intravenous hydration (1500–2000 mL/day), Urinary alkalinization with sodium bicarbonate, Glutathione (1200 mg/day iv), Magnesium isoglycyrrhizinate (200 mg/day iv). After 8 days of supportive therapy, CK decreased to 539 U/L (Figure 1A), myalgia improved to Grade I, and the patient was discharged off therapy. However, 13 days later, CK rebounded to 1735 U/L (Figure 1A) and myalgia worsened to Grade III. Supportive therapy was reinstated with the same therapeutic protocol as previously implemented. Pharmacotherapeutic efficacy evaluations were conducted on days 3, 7, and 11 after resuming supportive therapy. Within 7 days, myalgia ameliorated to Grade II, and CK levels demonstrated a declining trend (Figure 1A).

## 3 Discussion

Severe drug‐induced myopathy associated with trastuzumab T‐DXd is systematically reported here for the first time. T‐DXd comprises an anti‐HER2 antibody, cleavable linker, and topoisomerase‐I inhibitor DXd [11]. While known toxicities include interstitial lung disease (11.7%) and cardiotoxicity included QT interval prolongation (7.7%) and decreased ventricular ejection fraction (1.9%) [6]. In this case, the patient's cardiotoxicity biomarker NT‐proBNP showed mild elevation following T‐DXd therapy, while hs‐TnT remained negative (Table 1). After 2 cycles of T‐DXd treatment, left ventricular ejection fraction (LVEF) was preserved at 65.3% (Figure 3A) without concurrent decline despite CK elevation, inconsistent with the typical T‐DXd cardiotoxicity pattern [12, 13, 14]. The triad of biochemical‐morphological‐clinical concordance—elevated muscle enzymes, MRI‐documented edema, and neuromuscular symptoms—fulfills Level 1 diagnostic certainty for drug‐induced myopathy per European Neuromuscular Centre criteria [7, 15].

Mechanism on drug‐induced myopathy propose two primary pathogenic mechanisms: direct myofiber injury (e.g., mitochondrial or cytoskeletal damage) and immunologically mediated myopathy [8, 16]. In this case, the patient's antinuclear antibody (ANA) profile (Table 2), rheumatological markers, and inflammatory markers were unremarkable, effectively ruling out immune‐mediated myopathy.

Based on the phenotypic features of myopathy in this case, we performed a literature analysis to investigate the pathogenesis of T‐DXd‐induced myopathy. Current evidence suggests xenobiotic myotoxicity correlates with intracellular generation of superoxide radicals and hydrogen peroxide [17]. Functioning as critical bioenergetic hubs in skeletal muscle, mitochondria often develop impaired function following exposure to pharmacological toxins [18, 19, 20]. Rossi et al. [18] reported a 71‐year‐old patient with advanced lung adenocarcinoma who developed progressive proximal limb weakness and elevated serum CK following osimertinib administration. Myositis‐specific antibodies (MSAs) and myositis‐associated antibodies (MAAs) were negative. Muscle biopsy of the right biceps brachii revealed degenerative changes in myofibrils and mitochondrial dysfunction demonstrated by oxidative enzyme staining. Notably, statin‐induced drug‐related myopathy is also associated with mitochondrial dysfunction and dysregulation of cellular energy metabolism [19]. Research revealed that mitochondrial DNA exhibits high susceptibility to oxidative stressors including reactive oxygen species (ROS), where accumulation of ROS readily causes mitochondrial DNA damage [20]. The underlying mechanism of oxidative stress‐induced myocyte injury appears intrinsically linked to mitochondrial susceptibility in skeletal muscle tissue [18, 19, 20].

Irinotecan, a topoisomerase I inhibitor, and its bioactive metabolite SN‐38 promote oxidative stress accumulation and trigger DNA damage in colorectal carcinoma cells [21, 22]. Notably, irinotecan elicits intracellular ROS accumulation in human skeletal myocytes, which drives oxidative stress‐mediated damage to myotubes [23]. While T‐DXd myotoxicity remains undocumented, structural homology between DXd payload and irinotecan suggests potential shared oxidative injury mechanisms: (1) Direct mitochondrial injury by DXd: DXd exhibits high lipophilicity and membrane permeability (XLogP3‐AA 1) [24], readily accumulating in skeletal muscle mitochondria [25], which inhibits complex I activity, resulting in impaired ATP synthesis and reactive oxygen species (ROS) accumulation, thereby inducing muscle weakness and myalgia. (2) Linker instability: Approximately 2% of DXd undergoes premature release into circulation [3], directly damaging myocyte DNA. (3) Oxidative stress cascade: ROS burst triggers lipid peroxidation of myocyte membranes [26], leading to CK release into the bloodstream. Although recent studies associate high visceral adipose tissue (VAT) area with increased T‐DXd toxicity risk [27], this case occurred in a patient with normal body composition (VAT 69.1 cm^2^, skeletal muscle mass 23.9 kg), indicating myotoxicity can develop independently of body composition.

Following T‐DXd therapy, this patient developed elevated creatine kinase (CK 1755 U/L) and myalgia progressing to grade III, necessitating urgent therapeutic intervention. Existing evidence indicates that severe drug‐induced myopathy may culminate in rhabdomyolysis. When CK > 1000 U/L, the risk of renal injury significantly increases [28]. If respiratory muscles are affected, dyspnea may develop, further escalating to multi‐organ dysfunction and potentially fatal outcomes [29].

Current standard therapeutic approach consists primarily of glucocorticoid‐based immunosuppressive therapy [9]. In this case, immune‐mediated myopathy was ruled out, and the patient may not derive meaningful clinical benefit from corticosteroid intervention. Furthermore, studies indicate that high‐dose glucocorticoids may exert detrimental effects on disease progression in cancer patients [10]. This finding warrants cautious use of glucocorticoids in patients with advanced malignancies, particularly those receiving active antitumor therapy.

Integrating the observed biomarker elevations (CK/AST), we pursued an oxidative stress‐driven therapeutic approach for this case. A single‐center prospective randomized controlled trial demonstrated that magnesium isoglycyrrhizinate effectively prevents chemotherapy‐induced liver injury from oxaliplatin and capecitabine/5‐FU, reducing hepatic aspartate aminotransferase (AST) and alkaline phosphatase (ALP) levels [30]. Separately, another prospective RCT found that reduced glutathione combined with entecavir significantly decreased alanine aminotransferase (ALT) levels in chronic hepatitis B patients, improved liver function, and attenuated liver fibrosis [31]. Although systematic studies on magnesium isoglycyrrhizinate or glutathione for drug‐induced myopathy are lacking, existing evidence indicates that glutamine and glutathione supplementation may effectively treat severe myopathies [32]. Existing research indicated that glutathione directly scavenges reactive oxygen species (ROS), repairs oxidatively damaged sarcolemmal membranes, and inhibits CK release [33]. Magnesium isoglycyrrhizinate enhances antioxidant enzyme activities (superoxide dismutase, catalase, glutathione peroxidase), reduces malondialdehyde (MDA) levels and pro‐inflammatory cytokines, thereby exerting anti‐inflammatory, antioxidant, and anti‐apoptotic effects [34]. Within the oxidative stress cascade, ROS can induce lipid peroxidation, leading to the production of MDA [35]. Reduced glutathione and magnesium isoglycyrrhizinate alleviate oxidative stress damage by reducing MDA levels through distinct mechanisms: the former scavenges ROS [33], while the latter suppresses MDA generation [34]. Furthermore, magnesium isoglycyrrhizinate elevates the level of the antioxidant enzyme glutathione peroxidase [34], which catalyzes the neutralization of lipid peroxides and other oxygen free radicals by reduced glutathione [36], thereby exhibiting synergistic antioxidative effects. Cumulative evidence has validated that the combination of magnesium isoglycyrrhizinate and reduced glutathione confers substantial clinical efficacy for the management of chemotherapeutic agent‐induced acute liver injury [37].

The patient's biomarker profile—marked CK/AST elevation with incipient ALT rise—suggests glutathione plus magnesium isoglycyrrhizinate represents a mechanistically rational therapeutic approach with anticipated clinical efficacy. We administered the antioxidant agents glutathione and magnesium isoglycyrrhizinate to the patient, along with hydration and urinary alkalinization using sodium bicarbonate. Urinary alkalinization with sodium bicarbonate was implemented to mitigate the risk of myoglobinuric acute kidney injury (AKI) secondary to potential myoglobin precipitation.

Through the supportive regimen, we observed: (1) effective reduction in serum creatine kinase (CK) levels, (2) favorable trends in aspartate aminotransferase (AST) and alanine aminotransferase (ALT) values, and (3) alleviation of myalgia. The renal function remained stable within normal limits in this case. Based on these findings, we propose that glutathione and magnesium isoglycyrrhizinate may play a significant role in managing *T‐DXd*‐induced myopathy through their mechanistic rationale. Naturally, this therapeutic strategy requires robust validation through adequately powered clinical trials.

However, a critical caveat emerged: CK rebound occurred after discontinuation of supportive therapy, likely attributable to T‐DXd's prolonged half‐life and tissue retention effects [38]. Continuous administration is required until complete clearance of DXd (approximately 4 weeks). Clinically, it is imperative to establish a muscular toxicity management pathway and implement CK monitoring at baseline and during treatment to enable early intervention.

### 3.1 Limitations Statement

This single‐center case report has inherent limitations: uncontrolled design prevents confounder adjustment, limited follow‐up precludes long‐term assessment, and small sample size restricts generalizability. Findings should be considered hypothesis‐generating requiring RCT validation.

## Author Contributions

Treatment: Zhen Qiao, LiuDan Li, and Hong Wang. Financial support: LiTong Ye and Zhen Qiao. Administrative support: Hong Wang. Collection and assembly of data: LiTong Ye, Zhen Qiao, and ShuHui Dai. Manuscript Editing: LiTong Ye, Zhen Qiao, ShuHui Dai.

## Ethics Statement

This study was approved by the Ethics Committee of Zhuhai Maternal and Child Health Hospital (Approval Nos. V1.0‐2023011606 and V1.0‐2024(25)). The patient has provided signed informed consent.

## Conflicts of Interest

The authors declare no conflicts of interest.

## Data Availability

The original datasets generated during this case study are not publicly available due to patient privacy restrictions under institutional ethics committee regulations. De‐identified essential data supporting the key findings (including critical laboratory parameters, treatment response assessments, and summarized radiological evaluations) will be made available to qualified researchers upon reasonable request to the corresponding author. Requestors must provide an ethics‐approved study protocol and execute a legally binding data confidentiality agreement. Direct data inquiries to: Dr. LiTong Ye (Email: yelitong1@163.com).
